# 

**Published:** 1896-07

**Authors:** 


					﻿CONTENTS
ORIGINAL ARTICLES—
The Personal Equation in the Use of the Forceps. By Eugene
R. Corson, B. S., M. D., Savannah, Ga................ 319
Colles’s Fracture. By J. B. Morgan, M. D., Augusta, Ga.	326
Electricity in the Functional Neuroses. By A. D. Rockwell,
M. D., New York...................................... 332
SELECTIONS AND ABSTRACTS—
Enuresis and Its Treatment...........................   335
A Case of Gunshot Wound of the Stomach, with Three Perfora-
tions of the Same.................................... 338
Virulence of the Seminal Fluid in Secondary Syphilis... 343
Two Bicycle Dangers.................................... 345
Chronic Gastritis of Long Standing, with Periodic Attacks of
Migraine. Report of a Case........................... 347
SOCIETY REPORTS—
Richmond Academy of Medicine and Surgery ..... 849
EDITORIAL-
The Spitting Nuisance......................    355
NOTES...................   i...................... 357
BOOK REVIEWS.....................................  363
SPECIAL NOTES..................................    364
Prescriptions..................................... 870
				

